# Hydrophobic adsorbent prepared from spent methanol-to-propylene catalyst for directional adsorption of high COD oily wastewater

**DOI:** 10.1038/s41598-022-07766-4

**Published:** 2022-03-10

**Authors:** Xiaojing Yong, Hui Su, Nana Zhao, Zhengwei Jin, Min Yao, Yulong Ma

**Affiliations:** 1 Key Laboratory of High-Efficiency Utilization of Coal and Green Chemical Engineering, College of Chemistry and Chemical Engineering, Ningxia University, Yinchuan, 750021 Ningxia People’s Republic of China; 2 Institute of Coal Chemical Industry Technology, Ningxia Coal Industry Co., Ltd. of National Energy Group, Yinchuan, 750411 People’s Republic of China

**Keywords:** Chemistry, Energy science and technology, Engineering

## Abstract

Spent methanol-to-propylene (MTP) catalysts have a large specific surface area and high porosity but are usually disposed of in landfills directly, and recycling has rarely been reported. In this study, the spent MTP catalyst was moderately dealuminized with organic acids and etched with alkali solvent to increase its specific surface area, further silanized by octyl triethoxy silane (OTS). A novel superhydrophobic adsorbent covered with –Si(CH_2_)_7_CH_3_ groups was obtained. The characterization of XRD, SEM, FTIR and XPS shows that the adsorbent maintains a typical ZSM-5 zeolite structure, and the –Si(CH_2_)_7_CH_3_ group is successfully grafted into the sample, not only on the surface but also in some pore space. Taking high chemical oxygen demand (COD) wastewater as the object, the influence of contract time, pH and temperature on COD removal was investigated. The removal process could be better depicted by the Langmuir isotherm model and the pseudo second-order dynamic model. Furthermore, the results of the thermodynamic study (∆G is − 79.35 kJ/mol, ∆S is 423.68 J/mol K, and ∆H is 46.91 kJ/mol) show that the adsorption was a spontaneous and endothermic process. These findings indicate that the modified spent MTP catalyst has potential application for the removal of COD from wastewater.

## Introduction

MTP catalysts were formed by a ZSM-5 molecular sieve. The ZSM-5 molecular sieve has a unique pore structure that provides good ion exchange performance and shape selection selectivity^1,2^. Because of its good thermal stability, hydrothermal stability, wide distribution of Si/Al, and wide range of acid adjustability, ZSM-5 zeolite is a good solid acid catalyst and catalyst carrier^3,4^. It is widely used in the field of catalysis, such as in the petrochemical industry and coal chemical industry, as well as in the field of sewage purification and selective adsorption^5,6^.

Industrial wastewater contains many pollutants, among which organic compounds are one of the most toxic pollutants^7,8^. Zeolites are widely used for heavy metal ion removal^9–12^. It is necessary to have hydrophobic and oil-friendly adsorption of organic matter^13^. It is also important to improve the hydrophobicity of ZSM-5 by increasing the ratio of silicon to aluminum. However, the surface of ZSM-5 zeolite contains Si(Al)OH, which affects its hydrophobicity^14^. Generally, the static water contact angle (*θ*) is small and far from those of hydrophobic (θ > 90°) and superhydrophobic (θ > 150°) zeolites. This limits the partial application of ZSM-5 in hydrophobic catalysts and as a selective adsorbent. Modification of surfactant is an effective way to improve the hydrophobicity of a molecular sieve^15,16^. The hydrophobic group can be grafted onto the surface of the molecular sieve through the reaction of organosilane reagent with hydroxyl group. This improves its hydrophobicity and hydrophobic retention ability^15^.

Many published studies have reported that catalysts suitable for methanol to propylene (MTP) require small grain HZSM-5 molecular sieves with a high Si to Al ratio and a multistage pore composite structure^14,17,18^. Researchers have focused on the design and development of new MTP catalysts but rarely studied the comprehensive utilization technology of the functionalization of spent MTP catalysts. The spent MTP catalyst has a large specific surface area, high porosity, and good adsorption performance. There are several silicon aluminum hydroxyl groups on the surface, which have certain hydrophilicity and limit its application in oil-bearing wastewater. The process water, produced in the process of synthetic gas to oil or low carbon olefin from coal, is oily, and the COD content is high. It is necessary to pass the complicated oil–water separation process in the recycling process to ensure effective reuse of process water. Adsorption technology is often used in industrial plants to treat oil-bearing wastewater^7,19^. The selection and application of adsorbents is critical. In this paper, a spent MTP catalyst was modified with surface silane after acid–base treatment, and a superhydrophobic zeolite was obtained.

## Raw materials and preparation methods

### Raw materials

The raw materials were waste catalyst for methanol to propylene (sample: FMTP catalyst) in a fixed bed reactor, oxalic acid (H_2_C_2_O_4_), NaOH, HNO_3_ solution (68 wt%), OTS, deionized water, *p*-xylene, and *n*-hexane. The waste water is from the bottom of methanol recovery tower in Ningxia Coal Industry Co., Ltd. The detail composition for the waste water is depicted in Supplementary Figs. S1–S12.

### Preparation of hydrophobic adsorbent

First, 100 g of spent MTP catalyst powder (below 200 mesh) was added into H_2_C_2_O_4_ solution (4 mol/L) with continuously stirring, and heated at 95 °C for 4–6 h in a water bath. Next, the solution was centrifuged (5000 r/min, 3 min), washed with deionized water, and dried at 120 °C overnight in a blower drying box (sample: FMTP-4 mol/LH_2_C_2_O_4_). Then, an acid-treated sample was added to 0.2 mol/L NaOH solution (liquid to solid ratio 5:1). Mechanical stirring treatment was conducted at 95 °C for 2 h, followed by centrifuging for 3 min at the rate of 5000 r/min. The obtained upper waste catalyst mud was then redispersed in 250 mL of dilute HNO_3_ solution (1 mol/L) and stirred magnetically for 30 min to remove the residue. After washing with deionized water until neutral, the catalyst was dried at 120 °C overnight in a blower drying box and calcined at 600 °C for 6 h in a muffle furnace. The powder was pressed into pieces and screened to obtain a 14–30 mesh. Then the adsorbent product can be obtained after acid–base treatment (sample: FMTP-4 mol/L H_2_C_2_O_4_ + 0.2 mol/L NaOH).

**Figure 1:**
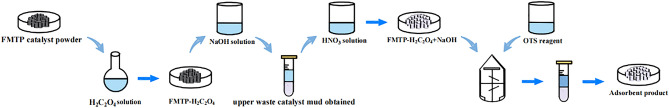
Flow chart of material modification.

40 g of adsorbent product was added to 80 mL of OTS reagent and placed in a 500 mL crystallization kettle. The reaction was performed at 120 °C for 10 h. After cooling, centrifugation was carried out, and the adsorbent was washed with absolute methanol 4–6 times. Vacuum drying was then conducted overnight at 70 °C to obtain a hydrophobic adsorbent (sample: Adsorbent product). The detailed flow chart of material modification is shown in Fig. 1.

### Analysis and characterization methods

The crystal structure of the samples was analyzed by X-ray diffraction (XRD, X-pert3 powder diffractometer, Panaco, Netherlands) with Cu Kα radiation (λ = 0.15406 nm) at a tube voltage of 40 kV and a current of 40 mA. The sample was recorded at a scanning rate of 5° min^−1^ over the 2θ range of 5°–80°. The framework structure of the samples was analyzed by Fourier infrared spectrometry (FTIR, a Bruker V70, Germany). The samples were prepared by mixing 1 mg of sample (1 wt%) with 99 mg of optical spectra grade KBr (99 wt%) in the wavelength range of 500–4000 cm^−1^, with a resolution of 4 cm^−1^. The Brunauer–Emmet–Teller (BET) specific surface area and pore properties of the samples were analyzed by a physical adsorption instrument (ASAP2020 surface analyzer, the United States). The samples were pretreated at 350 °C for 8 h before measurement. BET formula was used to calculate the specific surface area, DFT method was used to plot the pore size distribution curve, BJH method was used to calculate the mesoporous distribution, and T-plot method was used to calculate the pore volume. The chemical states and surface element contents of samples were investigated using X-ray photoelectron spectrometer (XPS, Thermo ESCALAB 250xi, United States) with Al Kα radiation. The composition of the samples was recorded via X-ray fluorescence spectroscopy (XRF, Bruker S8 tiger type, Zeiss Merlin, Germany). The micromorphology of the samples was observed using scanning electron microscope (SEM, Carl Zeiss, Germany), and the chemical environment of ^27^Al and ^29^Si elements in the samples was studied by nuclear magnetic resonance (NMR, electronic JNM ECZ600r NMR, Japan). Potassium dichromate method was utilized to measure the COD content through a COD analyzer (Hash, USA), and the hydrophilic/phobic properties was evaluated by measuring static water contact angle employing an OSA-100 contact angle goniometer (Rhoda Germany). Liquid chromatography–mass spectrometry technology (LC–MS, Clarus SQ8T, Perkin Elmer LTD) was utilized to analyze the composition of waste water. Temperature programmed GC Column: the initial temperature at 40 °C for 3 min, a processing temperature at 230 °C for 5 min at the rate of 10 °C/min. Carrier gas (constant current mode) at a flow rate of 1 mL/min. MS setting conditions: filament current of 10 mA, EI MASS spectrum, ion source temperature at 250 °C, solvent delay 2 min, ion scanning range of 40–500 m/z.

### Static adsorption experiment

Different weights of adsorbent (2 g, 4 g, 6 g, 8 g, and 10 g) and 100 mL of effluent containing different concentrations of COD (457.876 mg/L, 915.751 mg/L, 1831.503 mg/L, 3663.005 mg/L, 7326.01 mg/L and 12,210.017 mg/L) were added into a series of 250 mL iodine flasks. The wastewater mainly contained *n*-hexane, *p*-xylene, and methanol et al. After adjusting the pH, the effluent was placed at different temperatures (25 °C, 35 °C, 45 °C, 55 °C, and 65 °C) and oscillated for a certain time (30 min, 60 min, 90 min, 120 min, 180 min, 240 min, 270 min, and 300 min) in an oscillator to achieve adsorption equilibrium. Notably, when the adsorption rate and COD concentration were constant, the adsorption equilibrium was achieved. Finally, the COD concentration of wastewater was determined using the COD analyzer.

### Regeneration performance

The used hydrophobic adsorbent was calcined at 250 °C in a muffle furnace for 60 min to remove the adsorbed organic matter. After cooling down to room temperature, the obtained regenerated adsorbent can be reused in the wastewater for the removal of COD.

### Adsorption kinetics

Herein, the popular Lagergren equations, including Pseudo first-order and second-order, as well as the Elovich model have been used to analyze the experiment data, identifying the mechanism of COD removal by modified FMTP adsorbent. The detail equations are described as follows.

#### Pseudo first-order model

1 $${\text{lg}}\left( {{\text{q}}_{{{\rm e}}} - {\text{q}}_{{{\rm t}}} } \right) = {\text{lgq}}_{{{\rm e}}} - {\text{K}}_{{1}} {{\rm t}}$$

where *K*_1_ is the first-order adsorption kinetic constant (min^−1^), *q*_*e*_ is the adsorption amount in equilibrium (mg/g), and *q*_*t*_ is the adsorption amount at time *t* (mg/g).

#### Pseudo second-order model^10^

2 $$\frac{{\text{t}}}{{{\text{q}}_{{{\rm e}}} }} = \frac{{1}}{{{\text{K}}_{{2}} {\text{q}}_{{{\rm e}}}^{{2}} }} + \frac{{1}}{{{\text{q}}_{{{\rm e}}} }}{\text{t}}$$

where *K*_2_ is the quasi-second-rate constant of adsorption (min^−1^) and *q*_*e*_ is the adsorption amount (mg/g) in equilibrium.

#### Elovich model

3 $${\text{q}} = \frac{{1}}{{\text{b}}}{{\ln}}\left( {{\text{ab}}} \right) + \frac{{1}}{{\text{b}}}{\text{lnt}}$$

where a is the initial adsorption rate and 1/b is a parameter related to the number of adsorption sites.

### Adsorption isotherm

#### Freundlich isotherms^9,20^

4 $${\text{Inq}}_{{{\rm e}}} = {\text{InK}}_{{{\rm f}}} + \frac{{1}}{{\text{n}}}{\text{InC}}_{{{\rm e}}}$$

where *q*_*e*_ is the adsorption content of COD per unit adsorbent (mg/g) in equilibrium; *C*_*e*_ is the equilibrium concentration of COD in the solution (mg/L); *K*_*f*_ is the Freundlich isothermal constant (mg/g); and *n* is the adsorption strength (g/L).

#### Langmuir isotherms^9,20^

5 $$\frac{{1}}{{{\text{q}}_{{{\rm e}}} }} = \frac{{1}}{{{\text{q}}_{{{\rm m}}} }} + \frac{{1}}{{{\text{q}}_{{{\rm m}}} {\text{ K}}_{{{\rm l}}} {\text{ C}}_{{{\rm e}}} }}$$

where *q*_*m*_ is the maximum adsorption capacity (mg/g) and *K*_*l*_ is the Langmuir constant.

#### Dubinin–Radushkevich isotherms^9,21^

6 $${\text{Inq}}_{{{\rm e}}} = {\text{ Inq}}_{{{\rm m}}} - \upbeta \upvarepsilon ^{{2}}$$

where *β* is a constant related to the adsorption energy, and the *ε* value can be obtained from the following formula:

7 $$\upvarepsilon = {\text{RT In}}\left( {{1} + \frac{{1}}{{{\text{C}}_{{{\rm e}}} }}} \right)$$

where *R* is the gas constant (8.314 J/mol·K) and *T* is the absolute temperature (K).

### Adsorption thermodynamics

8 $$\Delta {\text{G}} = \Delta {\text{H}} - {\text{T}}\Delta {\text{S}}$$

9 $${\text{lg}}\left( {\frac{{{\text{q}}_{{{\rm e}}} }}{{{\text{C}}_{{{\rm e}}} }}} \right) = \frac{{\Delta {\text{S}}}}{{{2}{\text{.303R}}}} - \frac{{\Delta {\text{H}}}}{{{2}{\text{.303R}}}} \times \frac{{1}}{{\text{T}}}$$

In Eq. (9), *q*_e_ is the solid equilibrium concentration (mg/L) and C_e_ is the solution equilibrium concentration (mg/L). ΔS and ΔH in Eq. (8) are calculated by the slope and intercept of the Van’t Hoff isothermal formula.

## Results and discussion

### Adsorbent product characterization

**Figure 2:**
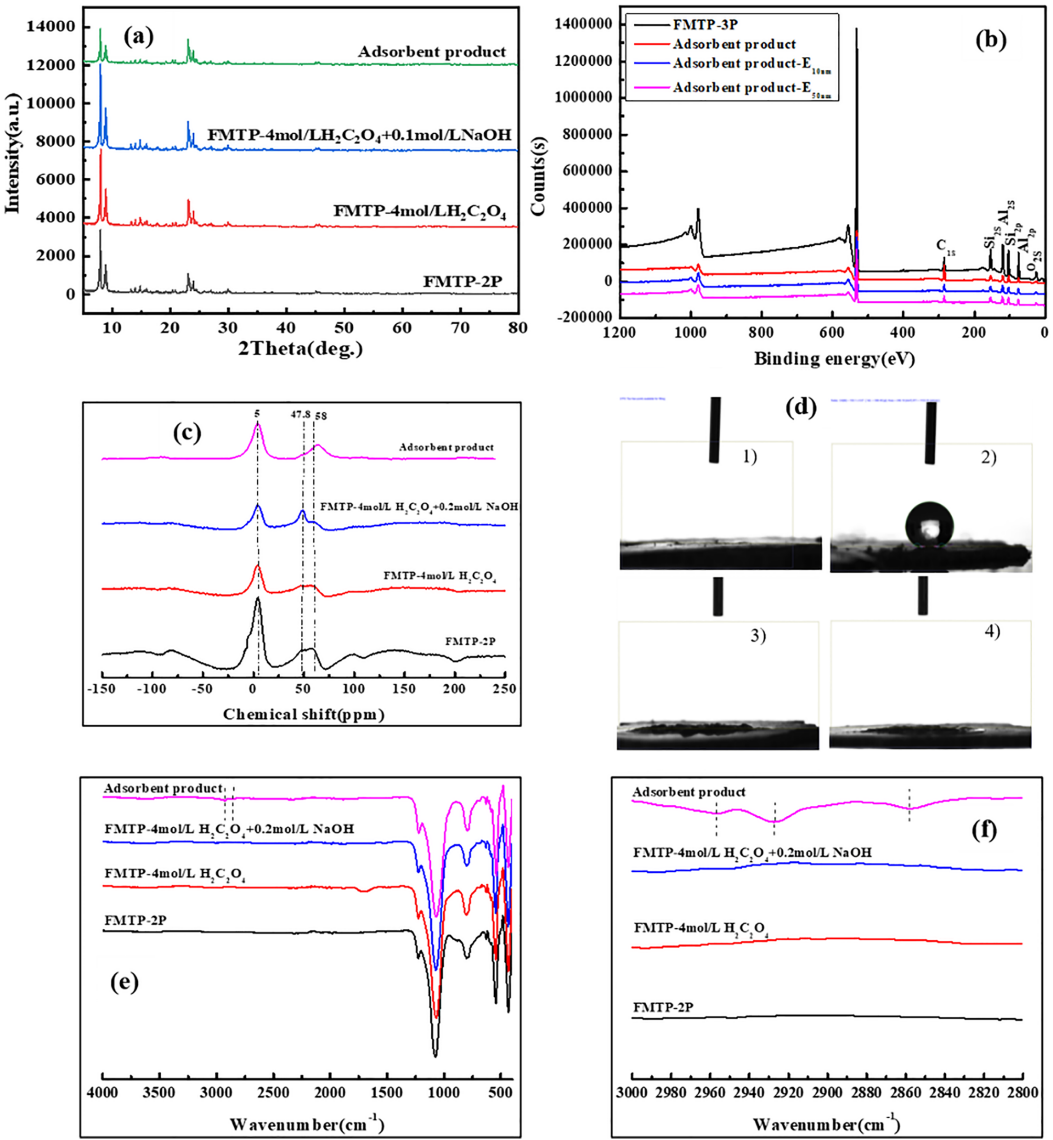
XRD of adsorbent products (**a**); XPS diagram of adsorbent products (**b**);^27^Al MAS NMR spectra of hydrophobic adsorbent product (**c**); Static CA of hydrophobic adsorbent products (**d**); FMTP-OTS without droplets (1), FMTP-OTS with water droplets (2), FMTP-OTS with *p*-xylene droplets (3) and FMTP-OTS with *n*-hexane droplets (4); Infrared spectrum of adsorbent products wavenumber from 500 to 4000 cm^−1^ (**e**), infrared spectrum of adsorbent products wavenumber from 2800 to 3000 cm^−1^ (**f**).

The XRD pattern of the adsorbent product is shown in Fig. 2a. The hydrophobic adsorbent product maintains a typical ZSM-5 molecular sieve structure, and the corresponding characteristic peaks corresponding to (011), (020), (051) and (033) crystal planes of ZSM-5 appear at 2*θ* = 7.9°, 8.8°, 23.1°, and 23.8°, respectively, indicating that the adsorbent channel has a long-range order^22–24^. It can be seen from Fig. 2b that the signals of C, Si, Al, and O elements in the samples before and after modification can be detected. The C 1s peak can be indexed to the air defiled carbon adsorbed on the surface of spent MTP catalyst, and all the samples show characteristic peaks of polluted carbon.

After modification with silane, the carbon content on the sample surface rises to 30.93%, which is attributed to the organic silane layer formed on the adsorbent surface. After the etching depth of the sample surface was extended from 10 to 50 nm, the carbon content decreases from 21.7 to 16.45%, demonstrating that the –Si(CH_2_)_7_CH_3_ group is successfully grafted onto the sample. The proposed surface reaction mechanism is as follows:

10 $${\text{Spent ZSM-5}} + {\text{H}}_{2} {\text{C}}_{2} {\text{O}}_{4} \to {\text{spent ZSM-5-H}} + {\text{C}}_{2} {\text{O}}_{4}^{2 - }$$

11 $${\text{Spent ZSM-5-H}} + {\text{NaOH}} \to {\text{spent ZSM-5-OH}} + {\text{H}}_{2} {\text{O}} + {\text{Na}}^{ + }$$

12 $${\text{Spent ZSM-5-OH}} + {\text{HNO}}_{3} \to {\text{ZSM-5}} + {\text{H}}_{2} {\text{O}} + {\text{NO}}_{3}^{ - }$$

13 $${\text{ZSM-5}} + {\text{OTS}} \to {\text{ZSM-5}} - {\text{Si}}({\text{CH}}_{2} )_{7} {\text{CH}}_{3}$$

Figure 2c shows the ^27^Al MAS NMR spectrum of the hydrophobic adsorbent product. Two kinds of aluminum species can be observed in the spent MTP catalyst. The peak at the general chemical displacement, *δ* = 50–60 ppm, belongs to the Al atom in the molecular sieve skeleton, which is tetrahedrally coordinated. The peak near *δ* = 0 is usually attributed to the hexahedral nonskeletal aluminum signal. There is a shoulder peak at *δ* = 47.8, which represents the signal of skeleton aluminum distributed on the outer surface of the molecular sieve or in a large hole. After modification of silane, the peak intensity of *δ* = 5.0 and *δ* = 58.0 increased, and the shoulder peak signal at *δ* = 47.8 disappeared, indicating that the –Si(CH_2_)_7_CH_3_ group has been grafted onto the hydrophobic adsorbent samples. And some pore space can be occupied, which was consistent with the BET results.

Figure 2d shows the static CA between the sample and water after silane modification. After OTS modification, the CA in Fig. 2d(1) reached 0° without water droplets, and when water dropped, the CA reached 159.1° [in Fig. 2d(2)], which shows a superhydrophobic state. It can be surmised that the surface of the sample is grafted with the low surface energy group –Si(CH_2_)_7_CH_3_, and the carbon chains are staggered to form a nanoscale rough surface, which improves the hydrophobicity of the sample surface. When *p*-xylene and *n*-hexane are dropped on the surface of the sample in Fig. 2d(3) and d(4), the droplets are immediately inhaled into the sample, indicating that the modified samples have strong adsorption to p-xylene and n-hexane.

Figure 2e and f shows the infrared spectrum of the adsorbent products. The ZSM-5 skeleton peak shows no obvious change after OTS modification, indicating that the skeleton structure of the adsorbent remains unchanged^25^. Absorption peaks at 1107 cm^−1^ and 804 cm^−1^ belong to the asymmetric expansion vibration peaks in the silicon oxygen tetrahedron, and the 457 cm^−1^ absorption peak indicates the Si–O–Si bending vibration. The absorption peak at 1230 cm^−1^ belongs to the asymmetric expansion vibration peak of silicon oxygen tetrahedron and aluminum oxygen tetrahedron in the ZSM-5 framework^10^. The absorption peaks of –CH_3_ and –CH_2_ appeared at 2859 cm^−1^, 2928 cm^−1^, and 2960 cm^−1^, respectively. This shows that the modified groups of –CH_3_ and –CH_2_ replace the Si–OH and –Si(CH_2_)_7_CH_3_ groups in the ZSM-5 skeleton and improve the hydrophobicity of the adsorbent.

The N_2_ adsorption and desorption curve and pore size distribution curve of the adsorbent products are shown in Fig. S1 (ESI). The adsorption capacity increased sharply when p/p_0_ < 0.05, indicating that there was microporous structure. The hysteresis ring appeared in the sample when p/p0 < 0.5, showing a characteristic type IV adsorption and desorption curve, indicating that the adsorbent product is a mesoporous composite material. Specific surface area and pore size analysis of the absorbent products are shown in Table 1. The specific surface area, pore volume, and pore size of the adsorbent after modification all decreased, suggesting that silane group grafting in the ZSM-5 molecular sieve pore occupies part of the inner space of the pore.

**Table 1:** BET specific surface area and pore structure parameters of adsorbent products.

Sample	S_BET_ (m^2^/g)	S_micro_ (m^2^/g)	S_meso_ (m^2^/g)	V_total_ (cm^3^/g)	V_micro_ (cm^3^/g)	V_meso_ (cm^3^/g)	Pore width (nm)
FMTP	304	106	198	0.29	0.05	0.24	3.77
FMTP-4 mol/LH_2_C_2_O_4_	356	96	260	0.33	0.05	0.28	3.68
FMTP-4 mol/LH_2_C_2_O_4_ + 0.2 mol/L NaOH	427	174	252	0.44	0.07	0.36	4.07
Adsorbent product	279	213	66	0.23	0.08	0.15	3.41

**Figure 3:**
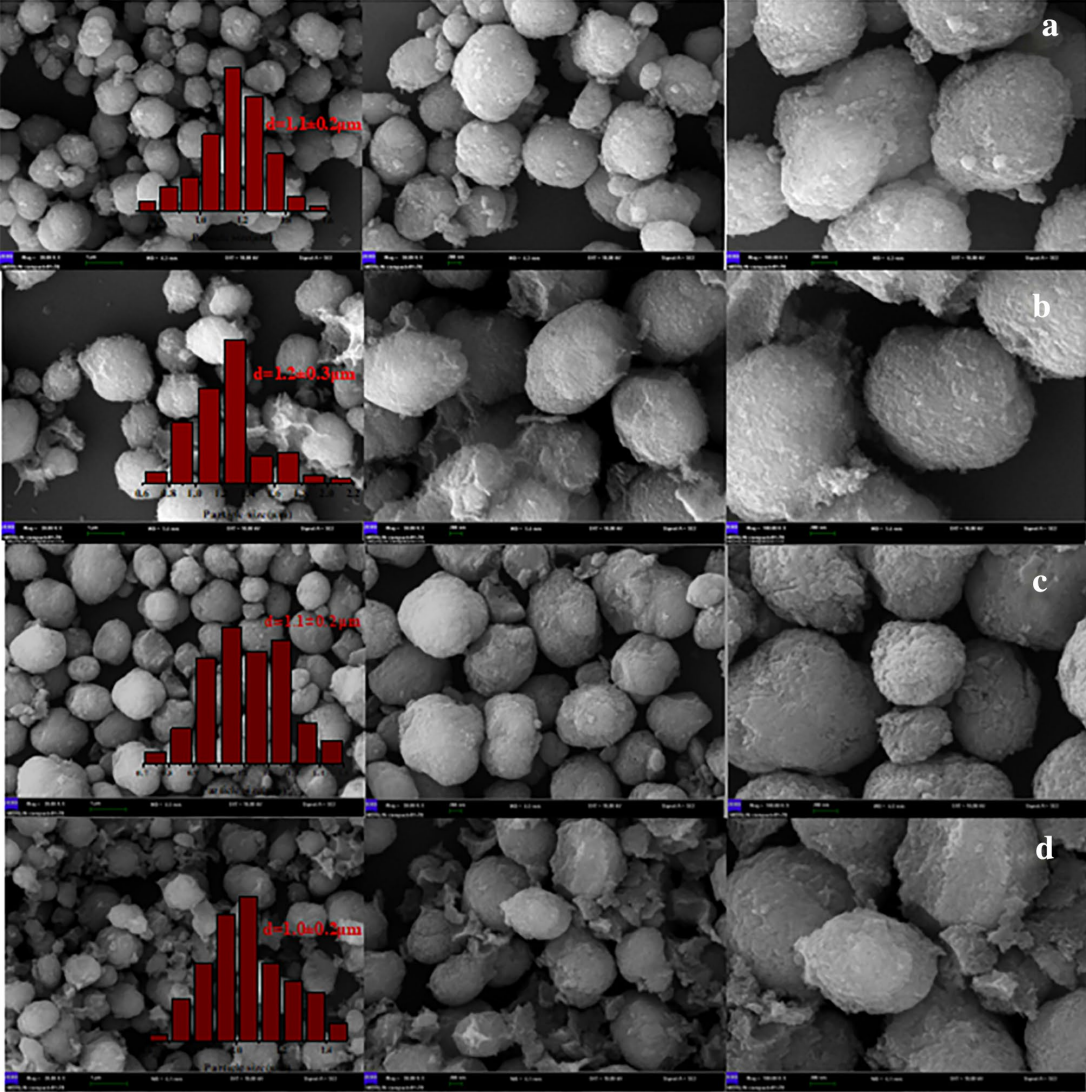
SEM of FMTP (**a**); FMTP-4 mol/L H_2_C_2_O_4_ (**b**); FMTP 4 mol/L H_2_C_2_O_4_ + 0.2 mol/L NaOH (**c**) and adsorbent product (**d**).

As shown in Fig. 3, the MTP catalyst sample after acid treatment shows an irregular ellipsoid shape with a uniform grain size of approximately 1.1 μm. After alkali treatment, the surface of the spheres became rough (Fig. 3c). However, the grain size for silane modified samples remained unchanged, and small aggregated particles appeared on the surface (Fig. 3d), testifying that the surface of the sample was loaded with silane groups.

As shown in Table 2, the ratio of Si/Al increases significantly after acid–base modification due to the increasing content of Si and decreasing content of Al element, which is similar to the Si/Al ratio in fresh catalysts. While the Si/Al ratio was almost unchanged after silane modification, certifying the composition of the adsorbent products was not affected by the silane modification. During the treatment of catalysts with different reagents, the elemental composition, except for Si and Al elements, show negligible change.

**Table 2:** Composition and content of adsorbent products.

Sample	Element component (wt.%)						SAR
	SiO_2_	Al_2_O_3_	Fe_2_O_3_	CaO	SO_3_	TiO_2_	
FMTP	79.02	20.64	0.0291	0.137	0.133	0.047	6.51
FMTP-4 mol/L H_2_C_2_O_4_	93.71	5.88	0.014	0.157	0.2	0.026	27.09
FMTP-4 mol/L H_2_C_2_O_4_ + 0.2 mol/L NaOH	93.85	5.81	0.012	0.139	0.164	0.025	27.46
Adsorbent product	93.62	6.03	0.015	0.172	0.136	0.027	26.35

### Effect of adsorption dose

**Figure 4:**
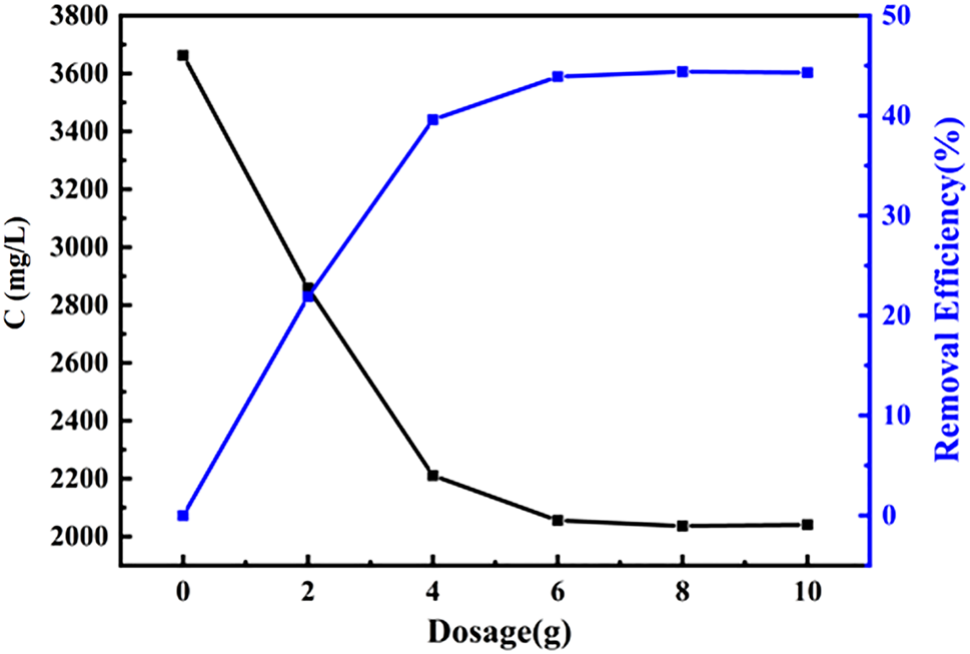
The curve of adsorption capacity and removal rate with the amount of adsorbent.

Particularly, the COD removal rate increases (in Fig. 4) gradually with the increasing dosage of adsorbent, which might be due to the large specific surface area. On the contrary, the content of adsorbed COD per gram of adsorbent shows a significant reduction with increasing adsorbent concentration. When the mass of adsorbent increased to 6 g, the COD adsorption capacity reached 156.3 mg and removal rate remained stable, suggesting the adsorption site reached saturation. Therefore, it can be determined that the optimum amount of adsorbent is 6 g, and the maximum removal rate of COD is 44.4%.

### Adsorption kinetics

**Figure 5:**
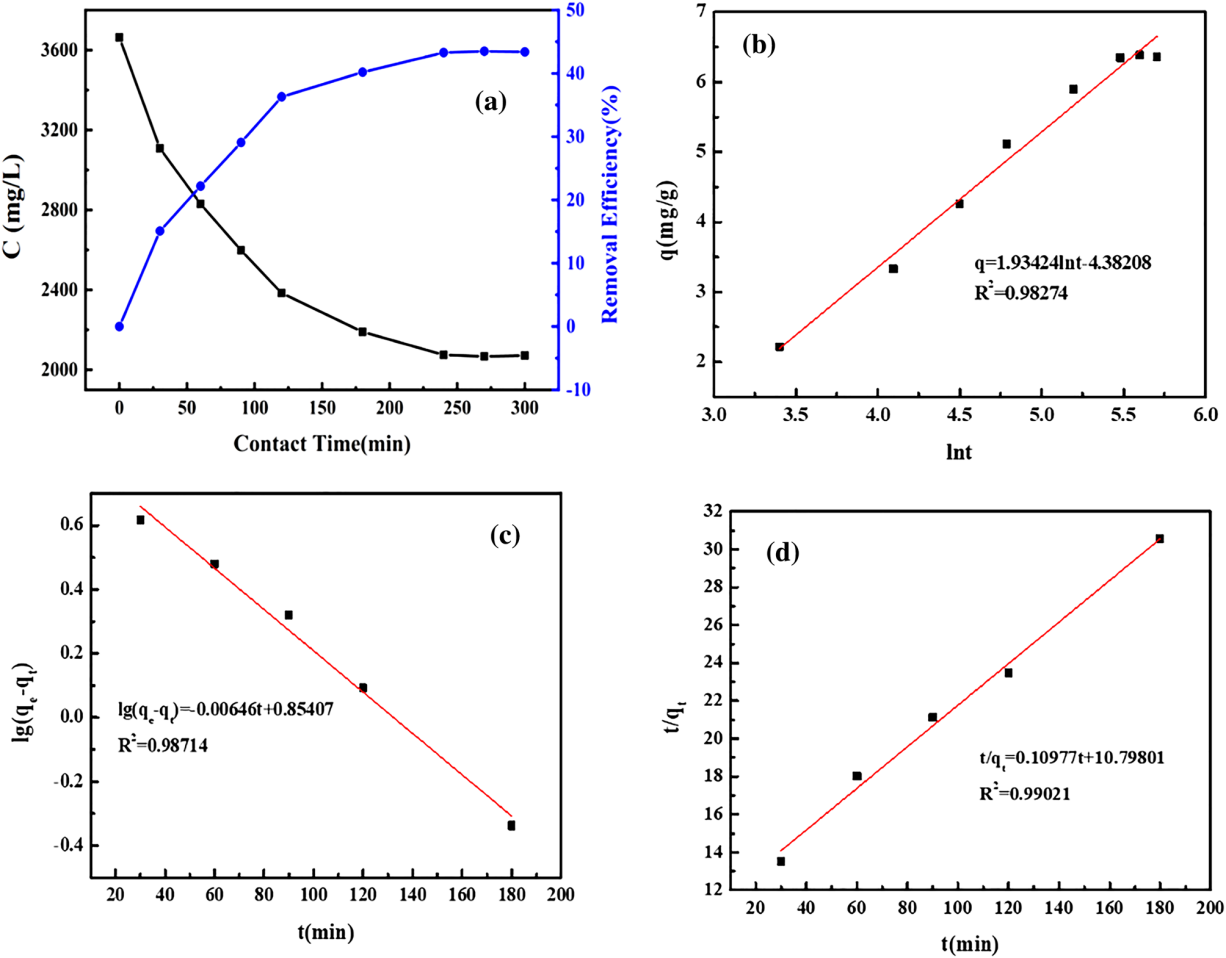
Curve of adsorption capacity and removal rate with time (**a**); Elovich dynamic model curve (**b**); Pseudo first-order dynamic model curve (**c**) and pseudo second-order dynamic model curve (**d**).

Here, the Elovich dynamic model has been used to investigate the adsorption kinetics shown in Fig. 5. The oil-bearing wastewater with the initial COD concentration of 3663.005 mg/L was adsorbed by the hydrophobic adsorbent (6 g). With the increased reaction time, the quantity of COD adsorbed (mg/g) and removal rate of COD in Fig. 5a showed a similar trend to that shown in Fig. 5. After 240 min of contact, the COD content remains constant, and the adsorption capacity reached equilibrium, indicating that the equilibrium between adsorbent (modified FMTP) and adsorbate (expressed as COD) was achieved. According to the pseudo first-order model in Eq. (1), pseudo second-order model in Eq. (2) and Elovich model Eq. (3), the kinetic constants can be obtained from the best fit equation shown in Fig. 5b and c. Although all the correlation coefficients (R^2^) obtained from the models are higher than 0.98, the pseudo second-order model may be agreeing with experimental values due to the chemical adsorption process^26^. The correlation coefficient (R^2^) in Fig. 5d is 0.99, demonstrating the presence of rate-controlling steps. Base on the above results, it can be concluded that the valence bond between adsorbent and adsorbate might be formed by sharing or swapping electrons^27,28^. The adsorption mechanism can be summarized as follows: (1) the adsorbate diffuses from the bulk solution to the surface of adsorbent; (2) the adsorbate penetrates into the pores inside the adsorbent; (3) the adsorbates are adsorbed on the active site of the adsorbent by sharing or swapping electrons^29^.

### Adsorption isotherm

**Figure 6:**
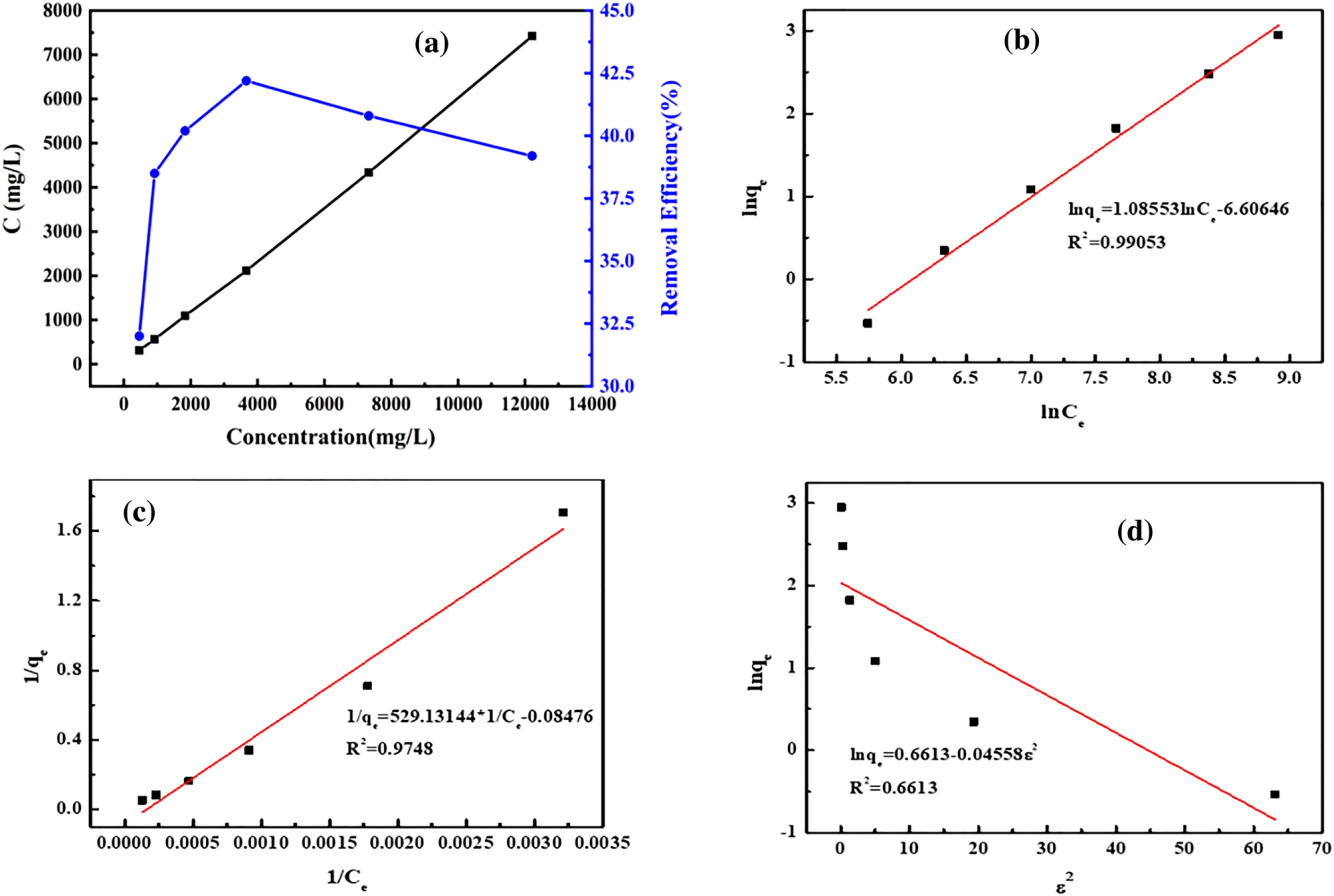
Variation curve of adsorption capacity and removal rate with concentration (**a**); Freundlich (**b**); Langmuir (**c**), and Dubinin–Radushkevich isothermal regression curve (**d**).

According to Eqs. (4)–(7), the adsorption capacity data in the Fig. 6a can be fitted with Langmuir, Freundlich, and Dubinin-Radushkevich isothermal regression curve models, which are shown in Fig. 6b–d. The plots of Freundlich (R^2^ = 0.990), Langmuir (R^2^ = 0.975), and Dubinin–Radushkevich (R^2^ = 0.661) isotherm model for COD removal are displayed in Fig. 6. By comparing the R^2^-values for the three models, it can be surmised that the Freundlich model, rather than Langmuir or Dubinin–Radushkevich model, could better explain the adsorption mechanism, suggesting that the active sites of the adsorbent showed different affinities for complex adsorbate in the effluent^30^. The similar result can also be found in another study for COD removal^26^. However, the fitted Langmuir model can still reveal the presence of monolayer adsorption on the surface of the adsorbent, which is not the dominant process.

### Adsorption thermodynamics and effect of pH

**Figure 7:**
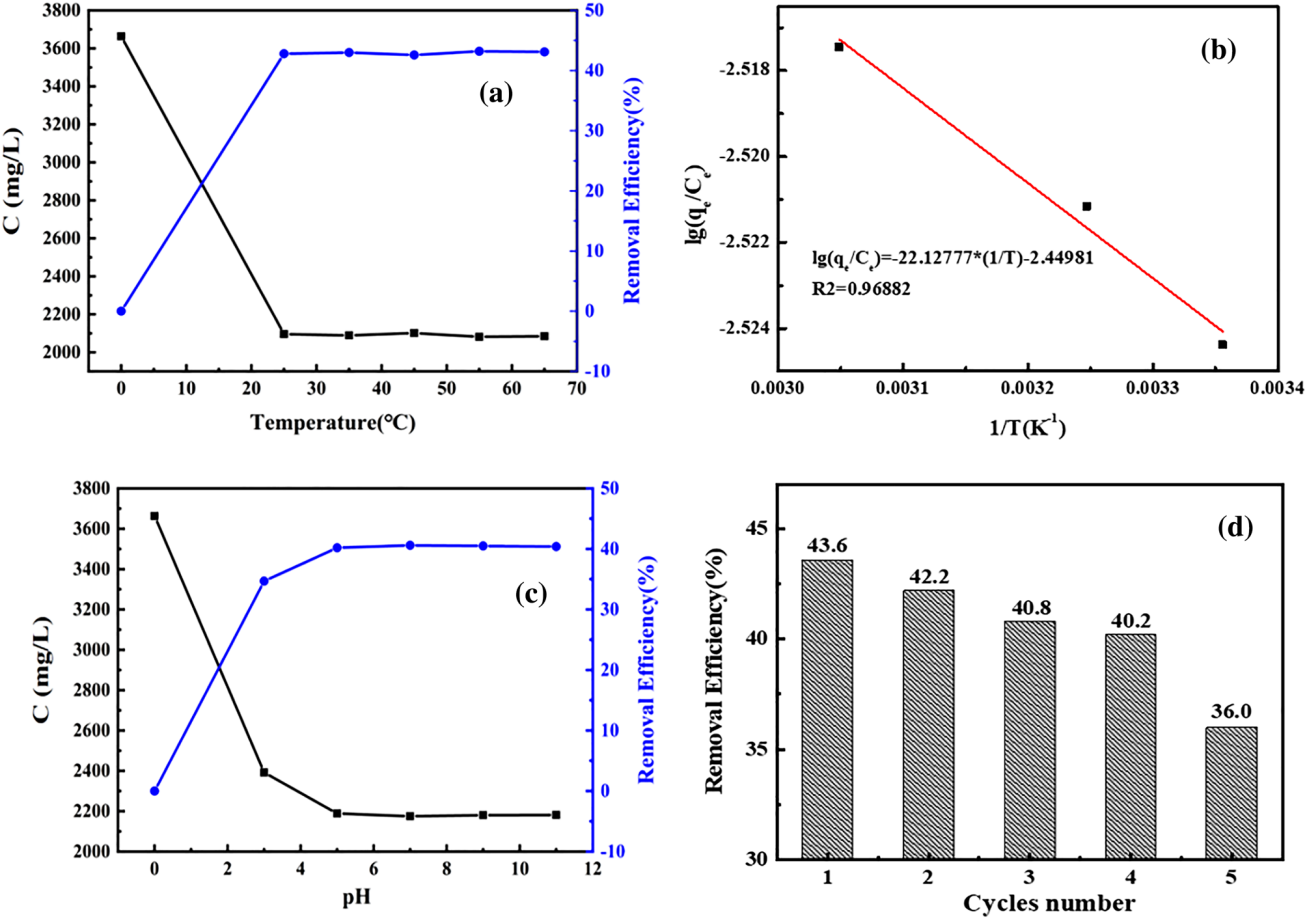
Variation curve of adsorption capacity and removal rate with temperature (**a**); thermodynamic curve (**b**); variation curve of adsorption capacity and removal rate with pH (**c**); COD removal efficiency by hydrophobic adsorbent after five cycles of regeneration (**d**).

Figure 7a shows the curve of adsorption capacity and removal rate with temperature. The results show that the adsorption capacity increases with the increasing temperature. When the temperature increased from 25 to 65 °C, the removal rate remained at 43.3%, certifying the increasing kinetic energy of the adsorbate is beneficial for the utilization of the active sites of the adsorbent. The thermodynamic curve obtained by fitting lg(*q*_*e*_/*C*_*e*_) to 1/*T* is shown in Fig. 7b. When the temperature is 298 K, the calculated thermodynamic parameters are 423.68 J/mol∙K for ∆S, − 79.35 kJ/mol for ∆G, and 46.91 kJ/mol for ∆H, proving that the adsorption process is spontaneous and endothermic. It can be observed from Fig. 7c that the solution at low pH value has a significant effect on the COD removal rate, and the COD removal rate increases slightly in the range of pH ≈ 4 to 10, which might be due to the destroyed –Si(CH_2_)_7_CH_3_ group under strong acid conditions^31–33^. The COD removal efficiency by hydrophobic adsorbent showed a marked decline in Fig. 7d after five adsorption cycles, which might be attributed by the incomplete desorption of adsorbate on the adsorbent surface. Considering the comprehensive cost, FMTP adsorbent that had been regenerated for four times is the best choice.

## Conclusions

In summary, a hydrophobic adsorbent was successfully synthesized using a spent MTP catalyst as the raw material. After treatment with acid and alkali, the spent MTP catalyst was modified using octyltriethoxysilane (OTS), and the –Si(CH_2_)_7_CH_3_ group was successfully grafted onto the surface of the adsorbent. The characterization of BET and XPS indicated that the silane group has been grafted on the surface and pore channel of the ZSM-5 zeolite and occupied the internal space of the pore channel. The adsorbent static water CA is 159.1°, and the hydrophobic adsorbent has a very strong adsorption performance for *p*-xylene and *n*-hexane. The maximum removal rate of COD in 3663.005 mg/L industrial wastewater by hydrophobic adsorbent is 44.4%. The adsorption kinetics can be described by a pseudo second-order kinetics curve due to the chemical adsorption process. The valence bond between adsorbent and adsorbate might be formed by sharing or swapping electrons. A speed-controlled step is existed in the adsorption process, which is spontaneous and endothermic. The adsorption isotherm type conforms to Freundlich isothermal regression curve model. In general, hydrophobic adsorbents obtained from spent MTP catalysts showed a great potential for the removal of high COD wastewater.

## Supplementary Information

Supplementary Figures.

## Data Availability

All data generated or analyzed during this study are included in this paper. Raw datasets are available from the corresponding author on reasonable request.
