# Supplementary material for: Hydrophobic adsorbent prepared from spent methanol-to-propylene catalyst for directional adsorption of high COD oily wastewater
Source: Sci Rep. 2022 Mar 10;12:3921. doi: 10.1038/s41598-022-07766-4 (PMC8913844; doi:10.1038/s41598-022-07766-4)
Supplement: Supplementary file 1 — Supplementary Figures. [file 41598_2022_7766_MOESM1_ESM.docx]

**Hydrophobic adsorbent prepared from spent methanol-to-propylene catalyst for directional adsorption of high COD oily wastewater**

Xiaojing Yong^a, b@^, Hui Su^b^, Nana Zhao^b^, Zhengwei Jin^b^, Min Yao^a@^, Yulong Ma^a@^

a. Key Laboratory of High-efficiency Utilization of Coal and Green Chemical Engineering, College of Chemistry and Chemical Engineering, Ningxia University, Yinchuan 750021, China

b. Institute of Coal Chemical Industry Technology, Ningxia Coal Industry Co., Ltd. of national energy group, Yinchuan 750411, China

@ **Corresponding authors** at: State Key Laboratory of High-efficiency Utilization of Coal and Green Chemical Engineering, College of Chemistry and Chemical Engineering, Ningxia University, Ningxia Yinchuan 750021, PR China

**E-mail address**: 377748087@qq.com (XJ. Yong), ndglym@163.com (M. Yao), yulongma796@sohu.com (Y.L. Ma)


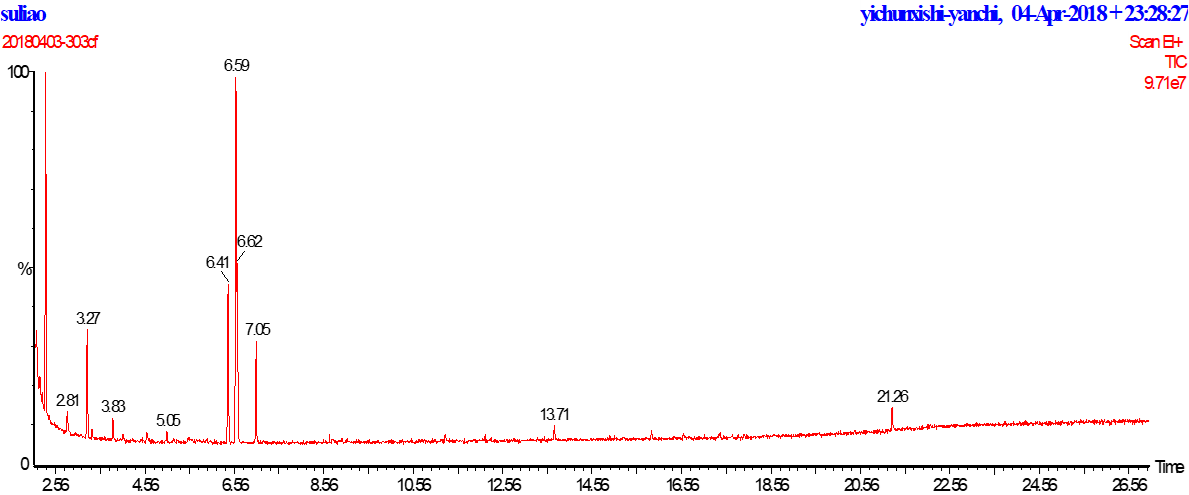


Fig. S1 Retention time of the waste water on LC-MS


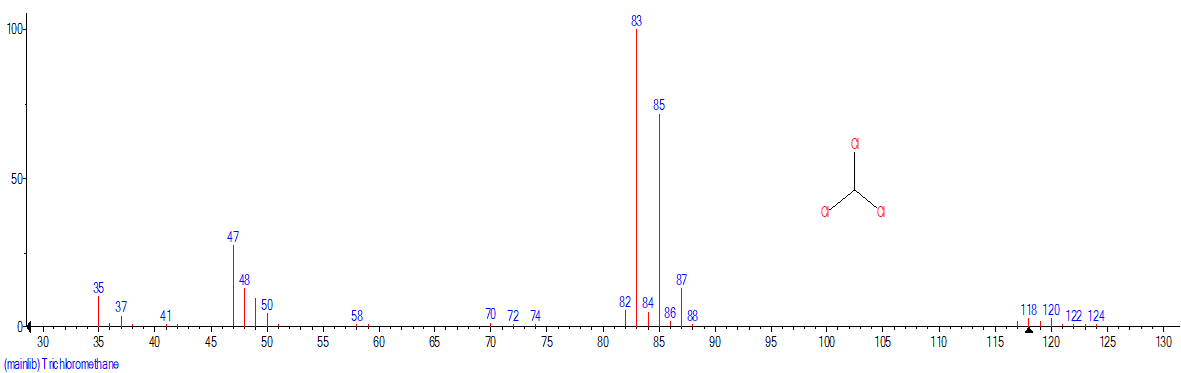


Fig. S2 LC/MS of Trichloromethane


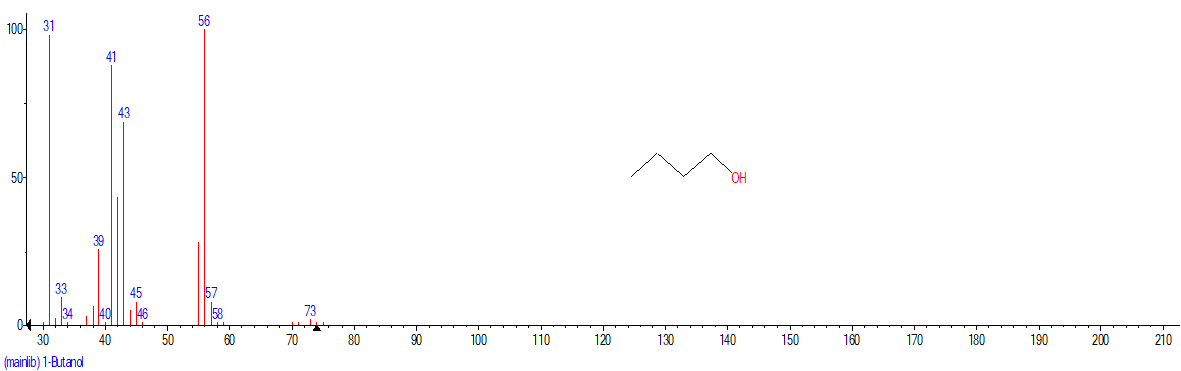


Fig. S3 LC/MS of 1-Butanol


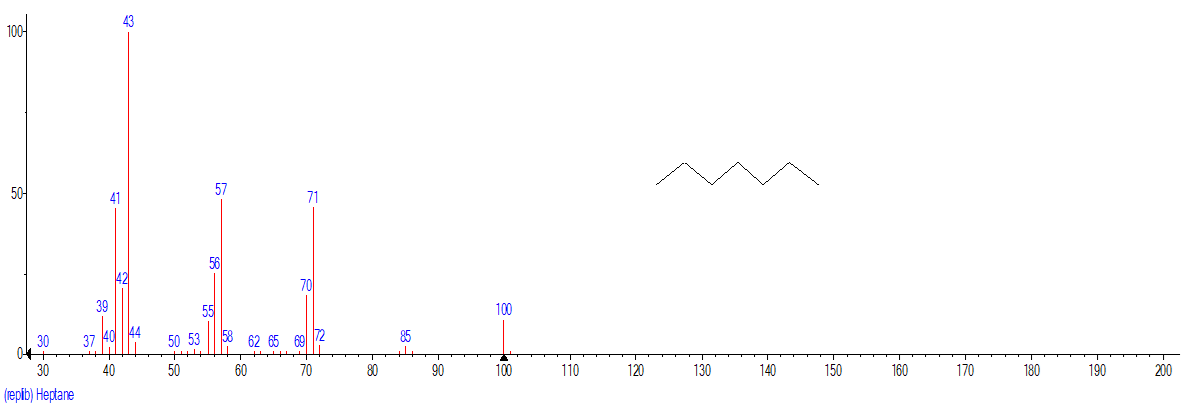


Fig. S4 LC/MS of heptane


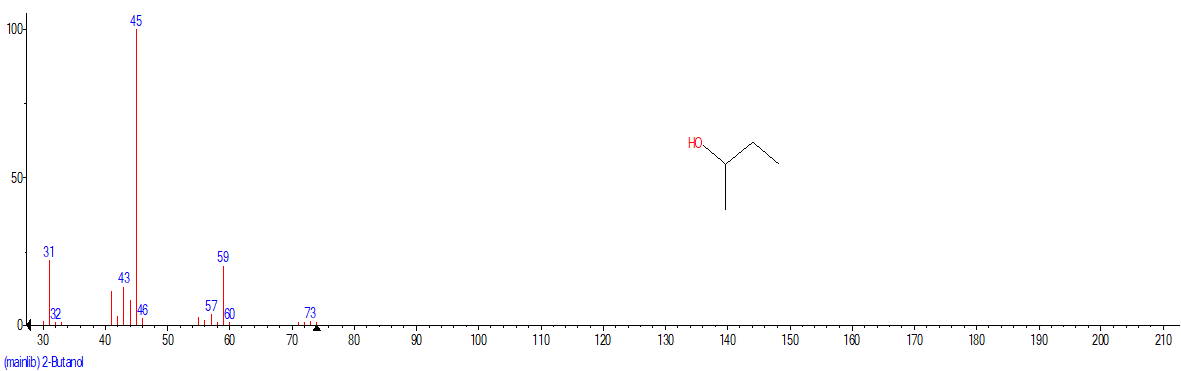


Fig. S5 LC/MS of 2-butanol


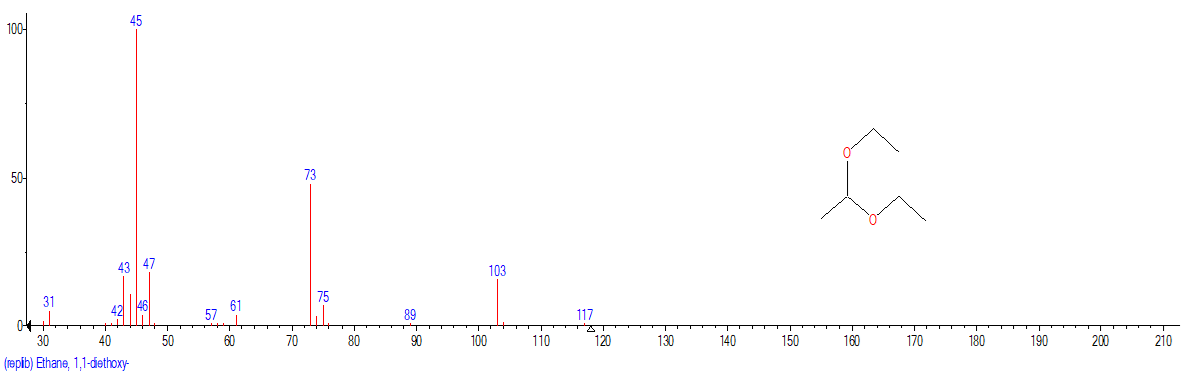


Fig. S6 LC/MS of 1,1-diethoxy-ethane


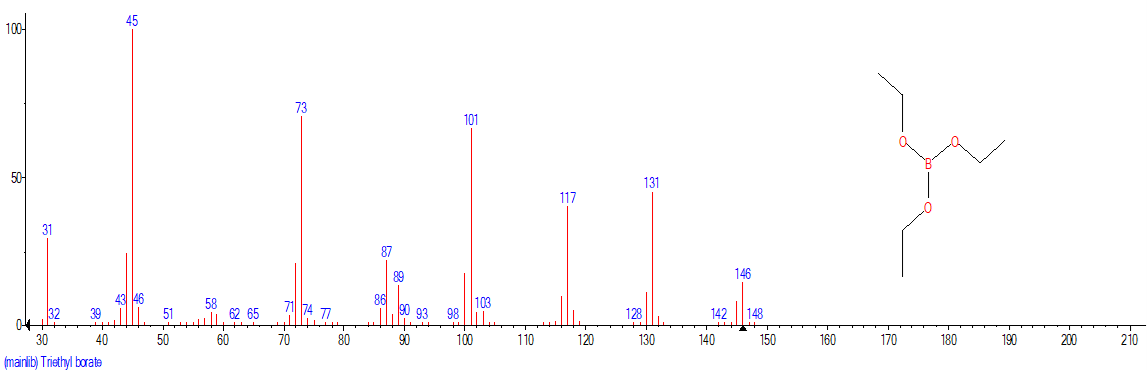


Fig. S7 LC/MS of triethyl borate


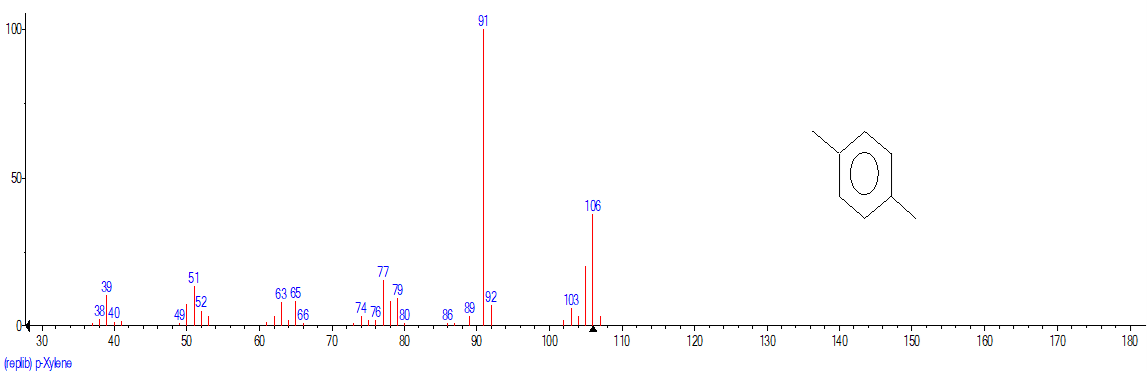


Fig. S8 LC/MS of p-xylene


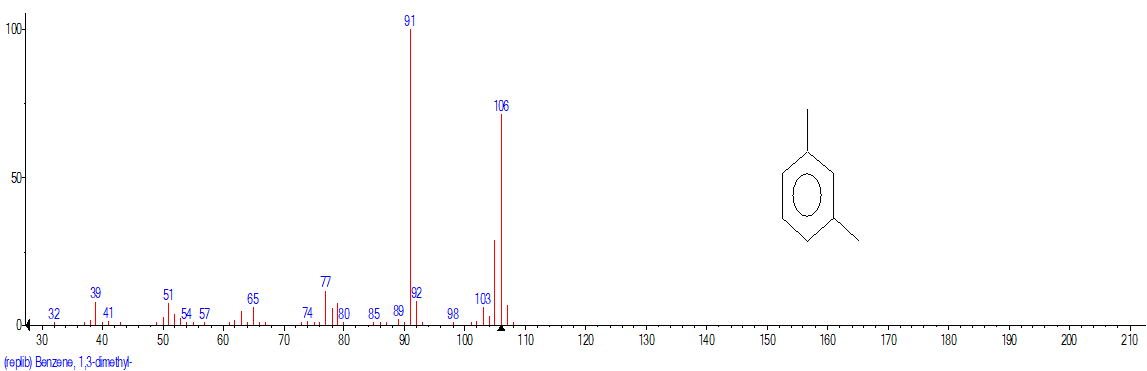


Fig. S9 LC/MS of 1,3-dimethyl-benzene


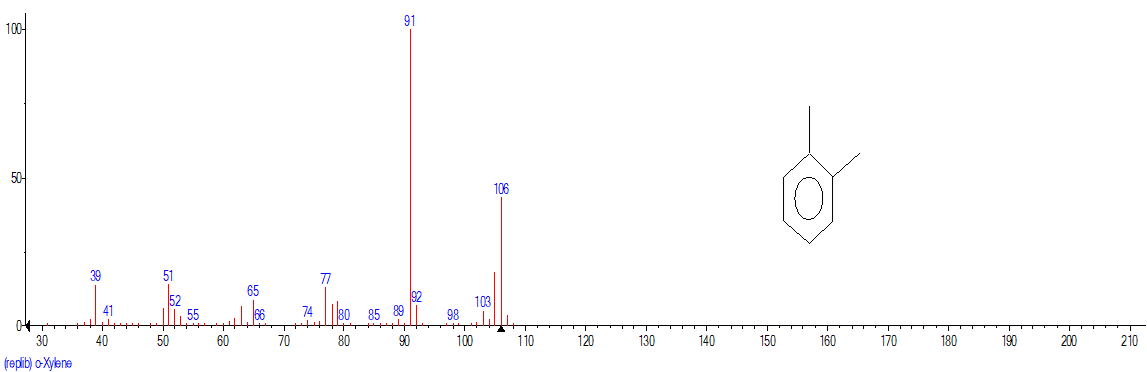


Fig. S10 LC/MS of o-xylene


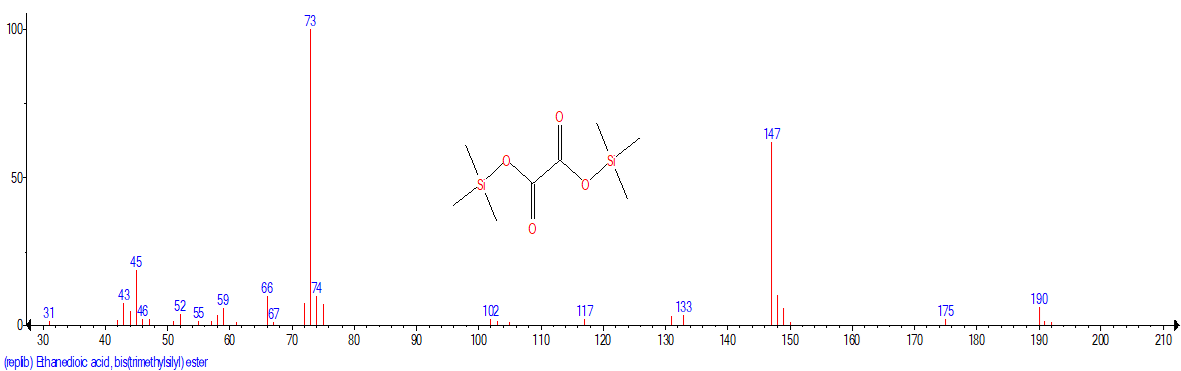


Fig. S11 LC/MS of ethanedioic acid, bis(trimethylsilyl) ester


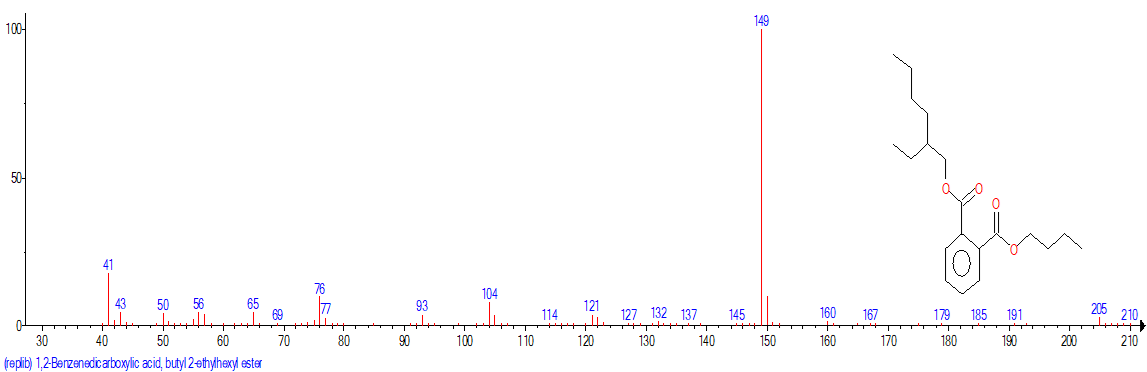


Fig. S12 LC/MS of 1,2-Benzenedicarboxylic acid, butyl 2-ethylhexyl ester


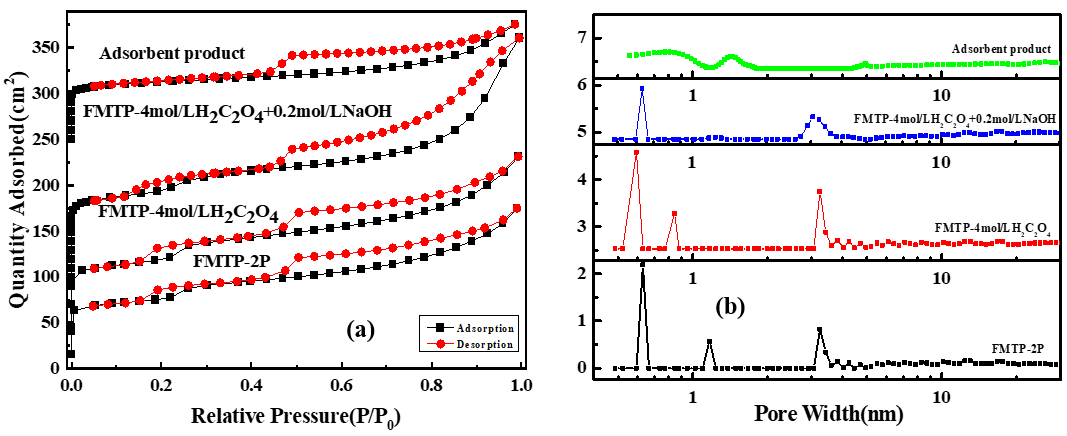


Fig. S13 Distribution of N_2_ adsorption/desorption isotherm (a) and pore size of adsorbent products (b)
